# The impact of lymphadenectomy on lymph node recurrence after performing various treatments for esophageal squamous cell carcinoma

**DOI:** 10.1186/s12893-022-01618-8

**Published:** 2022-05-11

**Authors:** Takashi Shigeno, Akihiro Hoshino, Shiho Matsunaga, Rumi Shimano, Naoya Ishibashi, Hajime Shinohara, Hiroyuki Shiobara, Chiharu Tomii, Katsumasa Saito, Naoto Fujiwara, Yuya Sato, Kenro Kawada, Masanori Tokunaga, Yusuke Kinugasa

**Affiliations:** Department of Gasrointestinal Surgery, Tokyo Medical and Dental University, 1-5-45 Yushima Bunkyo-ku, Tokyo, 113-8519 Japan

**Keywords:** Esophageal squamous cell carcinoma, Lymph node recurrence, Lymphadenectomy

## Abstract

**Background:**

Treatment for regional lymph node recurrence after initial treatment for esophageal squamous cell carcinoma (ESCC) differs among institutions. Though some retrospective cohort studies have shown that lymphadenectomy for cervical lymph node recurrence is safe and leads to long-term survival, the efficacy remains unclear. In this study, we investigated the long-term outcomes of patients who underwent lymphadenectomy for regional recurrence after treatment for ESCC.

**Patients and methods:**

We retrieved 20 cases in which lymphadenectomy was performed for lymph node recurrence after initial treatment for ESCC in our hospital from January 2003 to December 2016. Initial treatments included esophagectomy, endoscopic resection (ER) and chemoradiotherapy/chemotherapy (CRT/CT). Overall survival (OS) and recurrence-free survival (RFS) after lymphadenectomy were calculated by the Kaplan–Meier method. We also used a univariate analysis with a Cox proportional hazards model to determine factors influencing the long-term outcomes.

**Results:**

The five-year OS and RFS of patients who underwent secondary lymphadenectomy for recurrence after initial treatment were 50.0% and 26.7%, respectively. The five-year overall survival rates of patients who received esophagectomy, ER and CRT/CT as initial treatments, were 40.0%, 75.0% and 50.0%, respectively. The five-year OS rates of patients with Stage I and Stage II-IVB at initial treatments were 83.3% and 33.3%, respectively.

**Conclusions:**

Lymphadenectomy for regional recurrence after initial treatment for ESCC is effective to some degree. Patients with regional recurrence after initial treatment for Stage I ESCC have a good prognosis; thus, lymphadenectomy should be considered for these cases.

## Introduction

Esophageal squamous cell carcinoma (ESCC) is likely to spread to the cervical, mediastinal and abdominal lymph nodes through the lymphatic system [1]. In Japan, the recurrence rates after radical esophagectomy for ESCC are 29.0–43.0% [2–4]. Despite improvement of lymphadenectomy, lymph node recurrence accounts for 22.0–68.0% of all recurrence [2–7]. While some retrospective studies showed that the resection of regional lymph node recurrence had a good prognostic effect, the evidence was not strong [8–11].

As the adoption of endoscopic resection (ER) for superficial esophageal cancer became broader, more cases of lymph node recurrence after ER were reported [12–14]. Minasaki et al. suggested that the lymph node recurrence rate after ER of ESCC was approximately 4.0% [15]. The Japan Clinical Oncology Group (JCOG) reported a high complete response (CR) rate after radical chemoradiotherapy (CRT) for advanced ESCC (62.2%) [16]. However, their 5 year progression-free survival rate was no more than 25.6%, and lymph node recurrence after CRT for ESCC is common. In spite of the high recurrence rate, there have been few studies about the treatment of lymph node recurrence after ER and CRT.

In this study, we investigated the short- and long-term outcomes of patients who had lymphadenectomy for regional lymph node recurrence after treatment for ESCC.

## Patients and methods

### Data collection

We retrieved 20 cases of patients who underwent lymphadenectomy for regional lymph node recurrence after initial treatment for ESCC in our hospital from January 2003 to December 2016. We retrospectively collected the clinical data from patient records. This study was approved by Tokyo Medical and Dental University Medical Hospital Institutional Review Board (IRB number: M2021-267).

### Patients

Characteristics of 20 patients (male, n = 17; female, n = 3; median age, 67 years) who underwent lymphadenectomy for regional lymph node recurrence after initial treatment for ESCC are shown in Table 1. Table 2 showed that the characteristics of each patient who underwent lymphadenectomy for lymph node recurrence. The initial treatments for ESCC included had esophagectomy (n = 11), ER (n = 5) and CRT or chemotherapy (CT) (n = 4 [CRT, n = 3; CT, n = 1]).

**Table 1:** Characteristics of patients who received lymphadenectomy for lymph node recurrence

	Esophagectomy (n = 11)	ER (n = 5)	CRT/CT (n = 4)	Total (n = 20)
Age, median (range)	64 (56–84)	73(65–81)	65(56–73)	67(56–84)
Sex (%)				
Male	10 (90.9)	4 (80.0)	3 (75.0)	17 (85.0)
Female	1(9.1)	1 (20.0)	1 (25.0)	3 (15.0)
Location, Ce/Ut/Mt/Lt				
Ce	0 (0)	0 (0)	1 (25.0)	1 (5.0)
Ut	0 (0)	1 (20.0)	0 (0)	1 (5.0)
Mt	9 (81.8)	3 (60.0)	2(50.0)	14 (70.0)
Lt	2 (18.2)	1 (20.0)	1 (25.0)	4 (20.0)
Stage (%)				
I	2 (18.2)	5 (100)	0 (0)	7 (35.0)
II	2 (18.2)	0 (0)	1 (25.0)	3 (15.0)
III	4 (36.4)	0 (0)	0 (0)	4 (20.0)
IVA	2 (18.2)	0 (0)	1 (25.0)	3 (15.0)
IVB	1 (9.1)	0 (0)	2 (50.0)	3 (15.0)
Initial lymphadenectomy range				
Three-field	9 (81.8)	NA	NA	NA
The others	2 (18.2)	NA	NA	NA
pR0	10 (90.9)	5 (100)	NA	NA
Adjuvant therapy	4 (36.4)	0(0)	NA	NA
RFS, month, median (range)	13.4 (3.3–68.4)	50.5 (12.1–61.8)	19.9 (6.5–28.7)	15.9 (3.3–68.4)
Site of recurrence				
Cervical	9 (81.8)	3 (60.0)	1 (25.0)	13 (65.0)
Mediastinal	1 (9.1)	2 (40.0)	2 (50.0)	5 (25.0)
Abdominal	1 (9.1)	0 (0)	1 (25.0)	2 (10.0)
Approach of lymphadenectomy for recurrence				
Open	11	4	3	18
Mediastinoscopic	0	1	1	2

*ER* endoscopic resection, *CT* chemotherapy, *CRT* chemoradiotherapy, *pR* pathological residual tumor, *NA* not applicable, *RFS* relapse-free survival, *Ce* Cervical esophagus, *Ut* Upper thoracic esophagus, *Mt* Middle thoracic esophagus, *Lt* Lower thoracic esophagus

The stages of esophagectomy and ER are pathological. The stage of CRT/CT is clinical

**Table 2:** Characteristics of each patient who underwent lymphadenectomy for lymph node recurrence

Case	Age	Sex	Location	T	N	M	Stage	Site of lymph node recurrence	Recurrence outside of surgery and radiation area
Esophagectomy									
1	72	M	Mt	1b	0	0	IB	Rt. supraclavicular	Outside
2	77	M	Mt	1b	2	0	IIIA	Lt. supraclavicular	Inside
3	84	M	Mt	1b	3	0	IVA	Rt. supraclavicular Rt. cervical paraesophageal	Outside
4	64	M	Mt	1b	0	0	IB	Rt. cervical paraesophageal	Inside
5	59	M	Mt	3	2	0	IIIB	Lt. supraclavicular Lt. superficial cervical	Inside
6	75	M	Mt	3	1	0	IIIB	Cervical pretracheal	Outside
7	58	M	Mt	3	0	0	IIB	Rt. supraclavicular Rt. cervical paraesophageal	Inside
8	60	M	Lt	1b	1	0	IIB	Rt. cervical paraesophageal	Inside
9	56	F	Mt	3	2	1(LYM)	IVB	Lt. deep cervical	Outside
10	60	M	Mt	3	3	0	IVA	Pretracheal	Outside
11	75	M	Lt	3	1	0	IIIB	Paracardial	Inside
ER									
12	73	M	Mt	1b	0	0	IB	Rt. supraclavicular Rt. cervical paraesophageal	–
13	81	F	Mt	1b	0	0	IB	Middle thoracic paraesophageal Ligamentum arteriosum	–
14	65	M	Ut	1b	0	0	IB	Rt. supraclavicular	–
15	66	M	Mt	1b	0	0	IB	Rt. cervical paraesophageal	–
16	73	M	Lt	1a	0	0	IB	Rt. recurrent nerve	–
CT and CRT									
17	62	M	Ce	4	1	0	IVA	Lt. upper deep cervical	Inside
18	68	F	Mt	3	1	1(PUL)	IVB	Lt. recurrent nerve	Inside
19	56	M	Lt	2	0	0	II	Along the left gastric artery	Outside
20	73	M	Mt	3	1	1(LYM)	IVB	Rt. recurrent nerve	–

*M* male, *F* female, *Ce* cervical esophagus, *Ut* upper thoracic esophagus, *Mt* middle thoracic esophagus, *Lt* lower thoracic esophagus, *Rt* right, *Lt* left, *ER* endoscopic resection, *CT* chemotherapy, *CRT* chemoradiotherapy, *PUL* pulmonary, *LYM* lymph node

The stage in the Esophagectomy group and the ER group is pathological and the stage in the CT and CRT group is clinical

The disease stages (according to the 8^th^ edition of the UICC TNM Classification) of the 11 patients who received esophagectomy as an initial treatment were as follows: pStage I (n = 2), pStage II (n = 2), pStage III (n = 4), pStage IVA (n = 2), and pStage IVB (n = 1). All five patients who had ER had pStage I disease. Among four patients who had CRT or CT as initial treatment, one was cStage II, one was cStage IVA and two were cStage IVB. Among the 11 patients who had esophagectomy as an initial treatment, nine patients (81.8%) had three-field lymph node dissection. Among patients who received esophagectomy, 90.9% of patients achieved pathological residual tumor (pR) 0; among patients who received ER, this rate was 100%. All patients who had CRT or CT could achieve a clinical complete response (cCR) after initial treatment, based on the RECIST guidelines with endoscopic examination and enhanced computed tomography.

The median time from initial treatment to lymph node recurrence was 15.9 months. In cases involving esophagectomy and ER, the initial date was defined as the date of the operation or ER. In cases involving CRT or CT, we set the last date of CRT or CT treatment as the initial date. The median time from the initial treatment to lymph node recurrence was 13.4 months for esophagectomy, 50.5 months for ER and 19.9 months for CRT/CT. 13 patients (65.0%) had cervical lymph node recurrence, five (25.0%) had mediastinal lymph node recurrence, and two (10.0%) had abdominal lymph node recurrence. The median follow-up time after the resection of lymph node recurrence was 49.0 months.

The site of lymph node recurrence of the 11 patients who received esophagectomy as an initial treatment were as follows: cervical part (n = 9), mediastinal part (n = 1), abdominal part (n = 1). Among five patients who had ER as the initial treatment, three had the cervical recurrence and two had mediastinal recurrence. Among four patients who had CRT/CT as the initial treatment, one had the cervical recurrence, two had mediastinal recurrence and one had abdominal recurrence. Among five patients who had mediastinal lymph node recurrence, two patients had mediastinoscopic lymphadenectomy for recurrence.

### Exclusion criteria

We excluded cases of double cancer, with the exception of early-stage cancer, distant recurrence other than supraclavicular lymph node recurrence, and lymph node recurrence in multiple fields.

### Diagnosis of recurrence

The diagnosis of recurrence was based on imaging studies, including computed tomography and ^18^F-fluorodeoxy glucose emission tomography (FDG-PET).

### Lymphadenectomy for recurrence

When lymph node recurrence appeared within the area of initial lymphadenectomy, the enlarged lymph nodes were resected. When recurrence appeared outside of the area of initial lymphadenectomy and radiation and when ER and CT were performed as initial treatment, radical lymphadenectomy of the area including lymph node recurrence was performed.

### Statistical analysis

Statistical analyses were performed using the Stata/SE 16 software program. Overall survival (OS) and recurrence-free survival (RFS) were calculated by the Kaplan–Meier method. The two patients who had no metastatic lymph node on pathology after lymphadenectomy for recurrence were excluded from the survival analysis. A univariate analysis was performed using a Cox proportional hazards model. *P* values of < 0.05 were considered to indicate statistical significance.

## Results

The short-term outcomes of lymphadenectomy for recurrence are shown in Table 3. 17 patients (85.0%) achieved pR0. Histopathologically, seven patients had only one metastatic lymph node. Two patients had no metastatic lymph nodes. One patient had some cancer cells, but not in the lymph nodes. The rate of complications of Clavien-Dindo grade II or more was 30.0%, and that of Clavien-Dindo grade III or more was 15.0%; these included three cases of postoperative bleeding, lymphatic leakage and cervical abscess. The median hospital stay after lymphadenectomy was 4.5 days. The numbers of lymphadenectomy cases with perioperative therapy, according to initial treatment are shown in Table 4. 13 patients (85.0%) had perioperative treatment (e.g., CRT or radiation therapy [RT]) before and after lymphadenectomy for recurrence. Four patients (25.0%) had CRT as a neoadjuvant therapy. Three patients (15.0%) had RT as an adjuvant therapy, and six patients (30.0%) had CRT as an adjuvant therapy.

**Table 3:** The short- and long-term outcomes of lymphadenectomy for lymph node recurrence

	Esophagectomy (n = 11)	ER (n = 5)	CRT/CT (n = 4)	Total (n = 20)
Short-term outcomes				
Curability R0/R1/R2	9/1/1	4/0/1	4/0/0	17/1/2
No. of metastatic nodes, median (range)	2 (0–29)	2 (0–6)	5 (1–8)*	2 (0–29)
Postoperative hospital stay (days) median (range)	5 (2–26)	3 (3–15)	5 (3–13)	4.5 (2–26)
Complication (%) (Clavien-Dindo grade II ≤)	4 (36.4)	1 (20.0)	1 (25.0)	6 (30.0)
Long-term outcomes				
Five-year survival rate☨ (%) (95% CI)	40.0 (12.3, 67.0)	75.0 (12.8, 96.1)	50.0 (5.8, 84.5)	50.0 (25.9, 70.1)
Five-year relapse-free survival rate☨☨ (%) (95% CI)	25.0 (3.7, 55.8)	33.3 (0.9, 77.4)	25.0 (0.9, 66.5)	26.7 (8.7, 49.6)

*ER* endoscopic resection, *CT* chemotherapy, *CRT* chemoradiotherapy, *CI* confidence interval

*R* residual tumor

*We removed one patient who had some cancer cells, but not in the lymph nodes

☨We removed two patients who had no metastatic lymph nodes on pathology

☨☨We removed two patients who had no metastatic lymph nodes on pathology and three patients for whom R0 resection could not be achieve

**Table 4:** The number of lymphadenectomy cases with perioperative therapy

	Esophagectomy (n = 11)	ER (n = 5)	CRT/CT (n = 4)	Total (n = 20)
Neoadjuvant therapy CT/RT/CRT	0/0/4	0/0/0	0/0/0	0/0/4
Adjuvant therapy CT/RT/CRT	0/1/3	0/2/1	0/0/2	0/3/6

*CT* chemotherapy, *RT* radiation therapy, *CRT* chemoradiotherapy

**Fig. 1:**
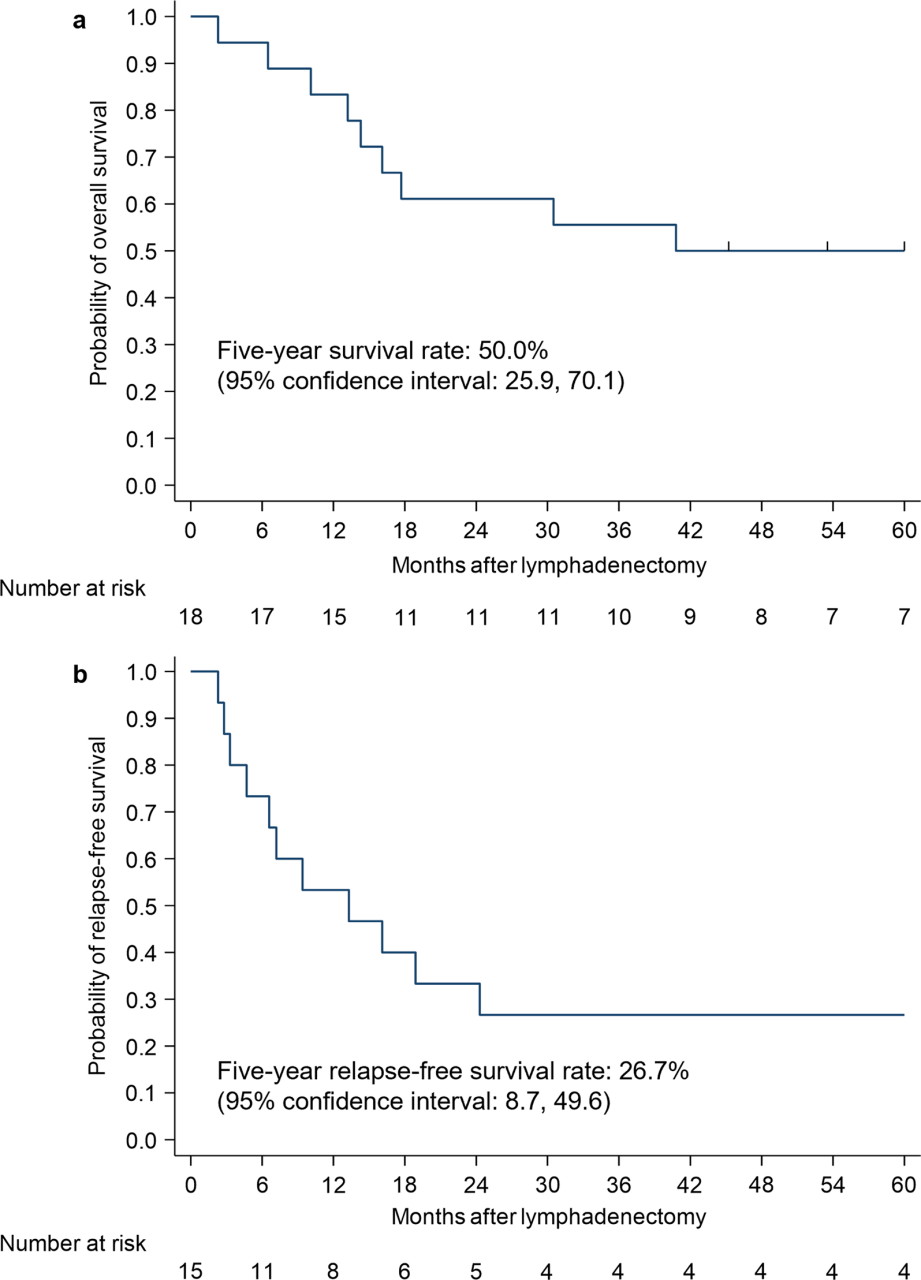
Kaplan–Meier estimates for patients who received lymphadenectomy. **a** Overall survival rate. **b** Relapse-free survival rate

**Fig. 2:**
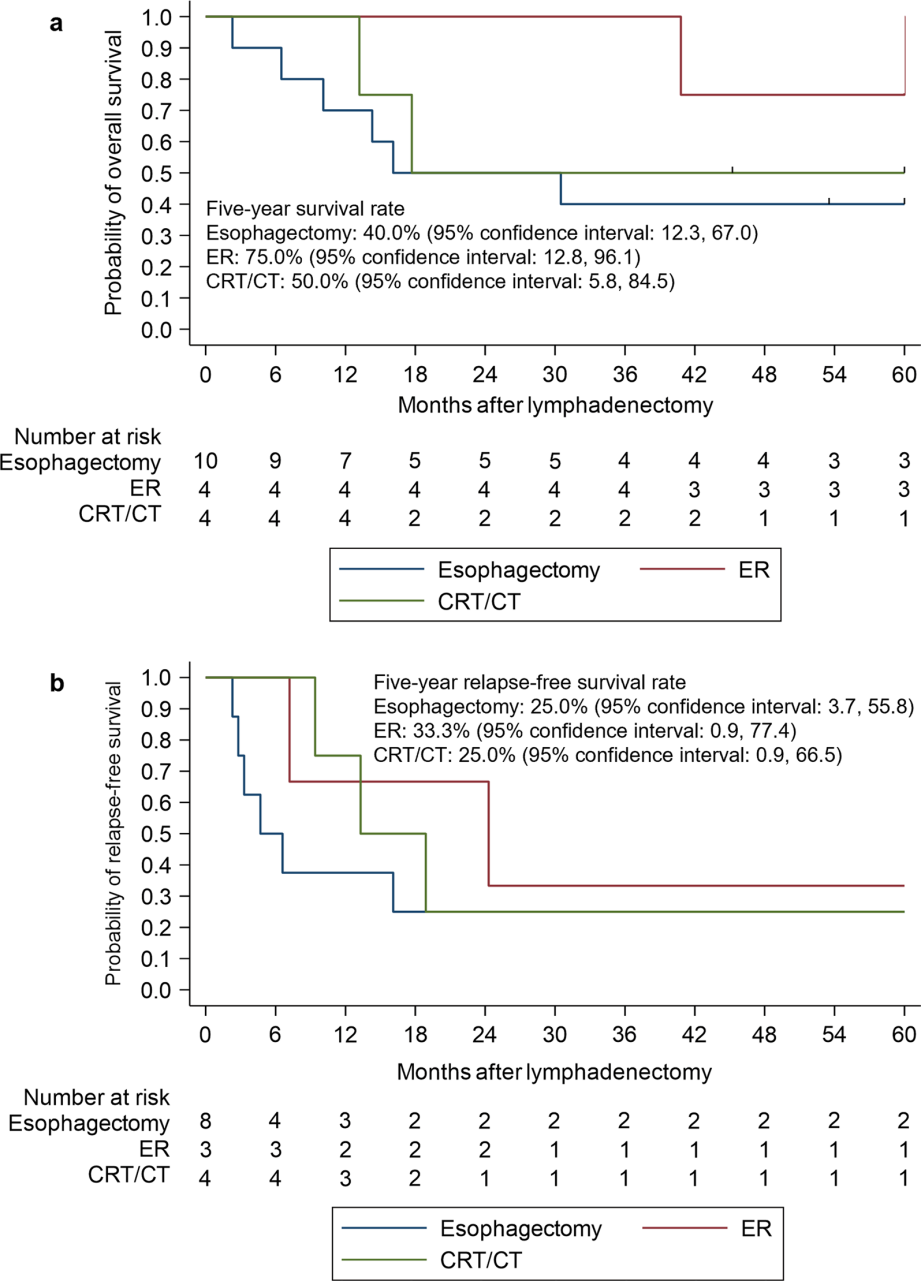
Kaplan–Meier estimates for patients who received lymphadenectomy, separated by initial treatment. **a** Overall survival rate. **b** Relapse-free survival rate. *ER* endoscopic resection, *CT* chemotherapy, *CRT* chemoradiotherapy

**Fig. 3:**
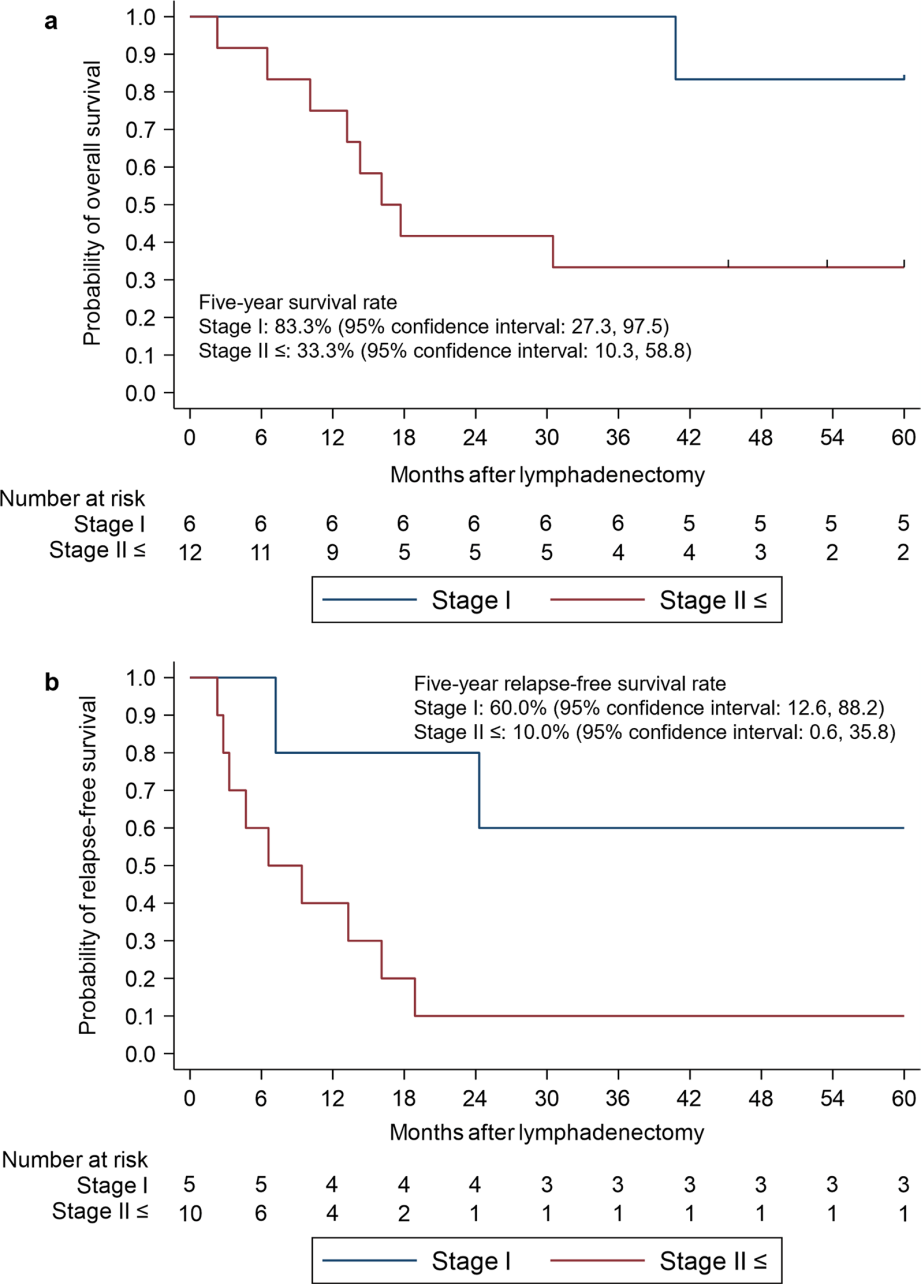
Kaplan–Meier estimates for patients who received lymphadenectomy, separated by stage. **a** Overall survival rate. **b** Relapse-free survival rate

The long-term outcomes of lymphadenectomy for recurrence are shown in Table 3. The five-year OS and RFS rates were 50.0% and 26.7%, respectively. According to the initial treatments, the five-year OS and RFS rates were 40.0% and 25.0%, respectively for esophagectomy; 75.0% and 33.3% for ER; and 50.0% and 25.0% for CRT and CT. These OS and RFS rates are also shown using the Kaplan–Meier method in Figs. 1 and 2. The results of the univariate analysis of overall survival with a Cox proportional hazards model are shown in Table 5. There were no significant differences in OS according to the initial treatment; the hazard ratios (HRs) for esophagectomy, ER and CRT/CT were 1.00, 0.20 (p = 0.14) and 0.59 (p = 0.52), respectively. When the locations of recurrence were compared, there were no significant differences in OS; the HRs for cases involving the cervical, mediastinal and abdominal areas were 1.00, 2.47 (p = 0.22) and 5.16 (p = 0.07), respectively. When cases in which pR0 was achieved were compared with those in which pR0 was not achieved, pR0 did not significantly improve the OS; the HR of pR1-2 was 1.49 (p = 0.62). When initial Stage I and Stage II-IVB were compared (Fig. 3), the HRs for OS and RFS in patients with Stage II or more disease were 4.27 (p = 0.08) and 3.57 (p = 0.07). We also examined the re-recurrence patterns after the resection of lymph node recurrence and showed the results in Table 6. We removed three patients who could not achieve R0. Among the six patients with initial Stage I, two patients had re-recurrence; they had regional lymph node recurrence. The two patients with regional lymph node recurrence included one patient who had re-recurrence in the right supraclavicular lymph nodes within the area of lymphadenectomy for recurrence. Among the 11 patients with initial Stage II or more disease, seven patients had re-recurrence; four patients had distant recurrence and three patients had regional lymph node recurrence. The three patients with regional lymph node recurrence included one patient who had re-recurrence in the left recurrent nerve lymph node s within the area of lymphadenectomy for recurrence.

**Table 5:** Results of the univariate analysis of factors associated with overall survival using a Cox proportional hazards model

Varieties	Hazard Ratio	95% Confidence Interval	p-value
Sex			
Male	1.00	Reference	Reference
Female	1.35	0.28, 6.50	0.71
Age (per year)	1.01	0.93, 1.09	0.86
No. of metastatic nodes	1.04	0.95, 1.13	0.41
Stage			
Stage I	1.00	Reference	Reference
Stage II ≤	4.27	0.84–21.76	0.08
Initial treatment			
Esophagectomy	1.00	Reference	Reference
ER	0.20	0.02, 1.66	0.14
CRT/CT	0.59	0.12, 2.86	0.52
Recurrent part			
Cervical	1.00	Reference	Reference
Mediastinal	2.47	0.59, 10.38	0.22
Abdominal	5.16	0.90, 29.5	0.07
pR			
0	1.00	Reference	Reference
1 ≤	1.49	0.31, 7.22	0.62

*ER* endoscopic resection, *CT* chemotherapy, *CRT* chemoradiotherapy, *pR* pathological residual tumor

We removed two patients who had no metastatic lymph nodes on pathology

**Table 6:** Patterns of re-recurrence after lymphadenectomy for lymph node recurrence

	Stage I (n = 6)	Stage II ≤ (n = 11)	Total (n = 17)
Distant recurrence	0 (0.0%)	4 (36.4%)	4 (23.5%)
Regional lymph node recurrence	2 (33.3%)	3 (27.3%)	5 (29.4%)
Within the area of lymphadenectomy for recurrence	1 (16.7%)	1 (9.1%)	2 (18.2%)
Out of the area of lymphadenectomy for recurrence	1 (16.7%)	2 (18.2%)	3 (17.6%)

We removed three patients who could not achieve R0

## Discussion

Some previous studies showed that the complication rate of lymphadenectomy for recurrence was 8.0–21.1% [8–11]. When these results are compared with our results, the complication rate in our study was slightly higher in comparison to previous studies. Four of six patients who had complications had esophagectomy, and three of them had CRT before lymphadenectomy for recurrence (lymphatic leakage, aspiration pneumonia and mediastinal abscess [n = 1 each]). We suspect that tissues were scarred and weakened from previous operations and CRT, which led to postoperative complications. We should carefully perform lymphadenectomy in cases after esophagectomy and CRT. The pR0 rate after lymphadenectomy for recurrence was nearly the same as the rate reported by Watanabe et al. (78.9%) and Nakamura et al. (88.2%) [8–10]. In addition, they reported median postoperative hospital stays of seven days and 10.5 days, respectively. In our study, the length of postoperative hospital stay was shorter in comparison to those previous studies.

Nakamura et al. reported that the median survival time of 22 patients who received CRT for lymph node recurrence after esophagectomy for ESCC was 20.3 months [10]. This study also showed that the rate of lymph node recurrence did not differ to a statistically significant extent between lymphadenectomy and CRT. In our study, the median survival time of patients who received lymphadenectomy for recurrence after esophagectomy for ESCC was 17.0 months. This result may suggest that the efficiency of lymphadenectomy for recurrence after esophagectomy for ESCC is limited. The combination of lymphadenectomy and adjuvant therapy may be needed at least, because eight of the 11 patients who received esophagectomy as an initial treatment also received CRT or RT.

There are few case reports about treatment for regional lymph node recurrence after ER for ESCC [12–14]. We performed lymphadenectomy for 5 patients with lymph node recurrence after ER for ESCC. Our study could not demonstrate that the prognosis of patients who received ER as an initial treatment for ESCC was significantly better in comparison to patients who had esophagectomy and CRT/CT; however, the results might imply the possibility that patients who received ER had a better prognosis. In addition, the interval from ER to lymph node recurrence was longer than from other initial treatments to lymph node recurrence. As Kanda et al. and Ono et al. reported lymph node recurrence two years after ER, regular and long-term follow-up are required to enable early intervention [17, 18].

In our study, we could not show a prognostic difference between initial Stage I and Stage II-IVB. However, patients with Stage I disease tended to show a better prognosis in comparison to those with higher disease stages. Ozawa et al. reported that the 5-year OS rates of patients with pStage I disease who had recurrence after esophagectomy for ESCC was 9.3%, which represents a poor prognosis [19]. Approximately 25% of the cohort of that study was composed of patients with distant metastatic recurrence; thus, we cannot simply compare the results with ours. However, the 5-year OS rate of patients with pStage I disease who had regional lymph node recurrence was 83.3%, which is a valuable result, even considering the unmatched cohort. Our results suggest that lymphadenectomy can lead to a good prognosis in patients diagnosed with pStage I ESCC who have regional lymph node recurrence.

The present study was associated with some limitations. This was a retrospective single-arm analysis of a limited number of cases. The indication of lymphadenectomy was based on the surgeons’ experience; thus, it is likely that there was a selection bias. We lacked information about some confounding factors, such as the performance status, the medical and social history, and the histopathological type. In addition, we could not perform a multivariate analysis because of the small study population. There is the possibility that we could not correctly assess the efficacy of lymphadenectomy itself because most of the patients who received lymphadenectomy also received some adjuvant therapy.

## Conclusions

Lymphadenectomy can be expected to be effective in the treatment of regional recurrence after the initial treatment of ESCC. In particular, patients with regional lymph node recurrence after initial treatment for Stage I ESCC had a good prognosis; thus, we should consider lymphadenectomy for these cases.

## Data Availability

The datasets generated and/or analyzed during the current study are not publicly available due to limitations of ethical approval involving the patient data and anonymity but are available from the corresponding author on reasonable request.

## Abbreviations

ESCC: Esophageal squamous cell carcinoma
ER: Endoscopic resection
CRT/CT: Chemoradiotherapy/chemotherapy
RT: Radiation therapy
OS: Overall survival
RFS: Relapse-free survival
JCOG: Japan Clinical Oncology Group
pR: Pathological residual tumor
cCR: Clinical complete response
FDG-PET: ^18^F-fluorodeoxy glucose emission tomography
HRs: Hazard ratios
PUL: Pulmonary
LYM: Lymph node
Ce: Cervical esophagus
Ut: Upper thoracic esophagus
Mt: Middle thoracic esophagus
Lt: Lower thoracic esophagus
CI: Confidence interval
